# AI-Driven Radiomics Assisted Prognostic Modeling for Hepatocellular Carcinoma with Portal Vein Invasion: A Retrospective Study

**DOI:** 10.3390/biomedicines14091894

**Published:** 2026-08-25

**Authors:** Tao Zhang, Xue Li, Yingli Guo, Junsong Zeng, Maosen Xu, Yan Tie

**Affiliations:** State Key Laboratory of Biotherapy, West China Hospital, Sichuan University, Chengdu 610041, China; zhangtao666@scu.edu.cn (T.Z.); 2020324020106@alu.scu.edu.cn (X.L.);

**Keywords:** hepatocellular carcinoma, radiomics, prognostic model, portal vein invasion

## Abstract

**Background**: Portal vein tumor thrombus (PVTT) marks advanced hepatocellular carcinoma (HCC) and carries a dismal prognosis. Survival varies widely even within this stage, yet simple tools for individualized risk stratification remain scarce. **Methods**: We retrospectively enrolled 134 HCC patients with PVTT and randomly divided them into a training set (*n* = 94) and a validation set (*n* = 40). Clinical predictors were selected by variance inflation factor screening and backward elimination Cox regression. A radiomics score (Rad-score) was constructed from portal-venous phase computed tomography (CT) images using Least Absolute Shrinkage and Selection Operator (LASSO) Cox regression with 10-fold cross-validation. Three Cox models were built: a clinical model, an imaging model based solely on the Rad-score, and a combined model integrating both. Discrimination was assessed by C-index and time-dependent area under the curve (AUC). Calibration was examined with bootstrap-based calibration curves. Decision curve analysis evaluated net benefit. A nomogram was developed from the combined model. **Results**: Four clinical variables (alpha-fetoprotein (AFP), body mass index (BMI), high-density lipoprotein cholesterol (HDL-C), and alkaline phosphatase (ALP)) and two CT texture features (GLRLM_SRHGE and GLZLM_SZHGE) were retained as independent predictors. The combined model gave the highest C-index in both the training set (0.843) and the internal validation set (0.815). Its 1-year AUC reached 0.953 and 0.947 in the two sets. Calibration slopes ranged from 1.044 to 1.291 across time points, indicating a tendency toward mild overdispersion; nevertheless, decision curve analysis confirmed net benefit across clinically relevant thresholds. The combined model offered greater net benefit than either single-domain model across a 0–50% threshold range. A nomogram incorporating all five predictors was generated for individualized 12- and 24-month survival prediction. **Conclusions**: A combined model integrating routine laboratory variables and a CT-based radiomics score improved survival prediction over clinical or imaging models alone. The corresponding nomogram uses inputs from a basic blood panel and a single portal-venous phase CT, suggesting its potential as a low-cost prognostic stratification tool for HCC patients with PVTT, although external validation in prospective multicenter cohorts is required before clinical implementation.

## 1. Introduction

Liver cancer causes over 800,000 new cases and more than 750,000 deaths each year worldwide, accounting for 4.3% of incident cancers and 7.3% of cancer deaths [1]. Hepatocellular carcinoma (HCC) dominates among all liver cancer cases. It is well established that early-stage disease can be controlled through surgery, ablation, or systemic treatment, and the survival rate is also good. But when it comes to advanced stages, the vast majority of patients, even liver cancer specialists, feel helpless. Even with modern regimens that combine chemotherapy, targeted agents, and checkpoint inhibitors, survival stays stubbornly short in advanced disease [2,3,4]. Better risk tools are needed, not just for predicting outcomes but for matching patients to the right intensity of treatment.

In clinical practice, most patients with portal vein tumor thrombus (PVTT) or extrahepatic spread fall into Barcelona Clinic Liver Cancer (BCLC) stage C [5,6,7]. The thrombus itself drives intrahepatic dissemination, decompensation, portal hypertension, ascites, and variceal bleeding [8,9]. Cancer progression is rapid, but treatment options are limited. Luckily, doctors have found that some patients survive considerably longer than the stage labels imply, but we lack simple bedside tools to distinguish them. If those who may benefit from more aggressive treatment measures can be identified at the initial stage of treatment, it will help improve the overall prognosis of the entire patient population. Of course, there are many factors that contribute to the differences in prognosis between different BCLC stage C patients.

We believe metabolism is part of the explanation, though not the whole story. The liver handles glucose, lipids, and proteins. HCC disrupts multiple important metabolic pathways. Insulin resistance, hyperglycemia, dyslipidemia, and hypoalbuminemia appear frequently, reflecting both tumor demand and hepatic reserve [10,11]. There is some evidence showing that metabolic syndrome has been tied to HCC risk, treatment response, and long-term outcome [12,13,14]. In advanced stages, chronic inflammation, malnutrition, and therapy-related stress also add further disruption [15,16,17]. We can consider that circulating metabolic markers carry dual information: they signal tumor burden and liver capacity at the same time. That makes them plausible prognostic candidates. Fasting glucose, triglycerides, albumin, and composite indices like triglyceride-glucose (TyG) have all been tested for prognostic value in HCC [18,19,20]. Some are independently associated with survival [21,22,23]. But any single marker captures only a slice of the metabolic picture. Combining several clinical variables is one way forward.

Another valuable direction for exploration is to look beyond bloodwork: artificial intelligence (AI) applied to routine computed tomography (CT) can extract quantitative features that the human eye misses. The core essence of tumors is still the pathological nature of the tumor itself, and imaging can partially display it in a macroscopic dimension. Radiomics extracts high-dimensional numerical descriptors of tumor shape, texture, and heterogeneity from standard CT or magnetic resonance imaging (MRI) [24,25,26]. These can be distilled into a radiomics score. In HCC, such scores have shown promise for predicting microvascular invasion, early recurrence, and treatment response [27,28,29]. What is less settled is whether adding a CT-based radiomics score to routine clinical and metabolic variables actually sharpens survival prediction in patients who already have PVTT. Answering that question would fit squarely into the push for AI-driven biomarkers in cancer care.

A model that combines metabolic variables, radiomics features, and standard clinical indicators has not been tested in HCC patients with PVTT. In this study, we attempted to build this model. Our goal was practical from the start: a tool that uses what is already collected—a blood test and a portal-venous phase CT—and returns individualized survival estimates. A more precise prognostic tool could eventually help guide treatment selection, including for aggressive therapies such as immunotherapy.

## 2. Materials and Methods

### 2.1. Patients Selection

Between January 2015 and December 2019, we pulled records of primary HCC patients who had undergone contrast-enhanced abdominal CT at West China Hospital. To be eligible, patients had to have pathologically confirmed HCC, CT evidence of PVTT, complete 5-year survival follow-up data, and routine laboratory tests within three months before CT. We excluded cases with indeterminate PVTT pathology, mixed tumor and bland portal vein thrombus, and any anti-tumor therapy—including transarterial chemoembolization (TACE), radiotherapy, targeted therapy, immunotherapy, or local ablation—within three months before imaging. We excluded patients whose last documented follow-up was shorter than six months from treatment initiation, as their survival status could not be reliably ascertained beyond the last known contact. Patients with complete event data—including those who died within six months—were retained in the cohort. The remaining patients were randomly split into a training set and a validation set. Hereafter in this manuscript, the term ‘validation set’ refers to this internal validation set derived from the same single-center cohort by random split, rather than an external validation set.

### 2.2. Data Collection

Demographic, clinical, imaging, and laboratory data were pulled from the hospital’s electronic medical record system, all obtained within three months before treatment. Lab tests were run on the hospital’s automated platform. Overall survival was measured from the date of first treatment until death from any cause or last follow-up.

### 2.3. CT Acquisition and Radiomics Workflow

Portal-venous phase CT images were used for radiomics analysis. All scans were performed using 64-detector row scanners of the same model following the center’s standard protocol. Detailed CT acquisition and reconstruction parameters, including contrast administration, scan timing, and imaging settings, are provided in Supplementary Material 1. A radiologist with experience in liver imaging manually segmented the primary tumor volume, avoiding vessels and necrosis. A second radiologist reviewed the contours, and disagreements were settled through discussion. Using LIFEx software (version 3.74; CEA-SHFJ, Orsay, France), we extracted a battery of shape, first-order, and texture features, as well as wavelet-transformed variants. All features were normalized to zero mean and unit variance using the training set’s statistics.

### 2.4. Radiomics Score Construction

In the training set, a LASSO Cox regression model, a supervised machine learning algorithm, was applied to the normalized radiomic features [30]. Ten-fold cross-validation selected the penalty parameter λ, and features with non-zero coefficients at that λ were retained. These features were linearly combined with their LASSO coefficients to form a radiomics score, or Rad-score. The same normalization parameters and linear formula were then applied to the validation set without modification.

### 2.5. Clinical Predictor Selection

We first screened clinical variables for collinearity using the variance inflation factor (VIF). Variables with VIF exceeding 5 were excluded from further analysis to address multicollinearity, which can destabilize coefficient estimates in Cox regression. The remaining variables then underwent univariate Cox regression, and those that met a relaxed significance level were carried forward into a multivariate Cox model. Backward elimination was then applied, retaining only predictors that remained significant at a conventional threshold. This procedure produced the clinical model.

### 2.6. Model Building and Evaluation

Three Cox proportional-hazards models were constructed in the training set. The clinical model contained the predictors retained after backward elimination. The Rad-score model contained only the continuous Rad-score. The combined model included all variables from the clinical model together with the Rad-score, entered simultaneously without further selection. The same model formulas were used in the validation set without refitting.

Discrimination was assessed with C-index and time-dependent AUC at two landmark time points, both in the training set and in the validation set. Calibration was assessed using the calibrate() function from the rms package with 200 bootstrap resamples. Calibration curves were generated by plotting observed survival probabilities against predicted probabilities, stratified by risk deciles. The calibration intercept and slope were derived from a linear regression of observed outcomes on predicted probabilities. Under this parameterization, an ideal intercept of 0 and an ideal slope of 1 indicate perfect calibration. An intercept > 0 indicates systematic underestimation of survival probabilities, while an intercept < 0 indicates systematic overestimation. A slope > 1 indicates overdispersion—predicted probabilities are too extreme—whereas a slope < 1 indicates underdispersion. Decision curve analysis was used to evaluate net benefit across a range of threshold probabilities. Risk scores from each model were dichotomized by the median of a time-dependent ROC curve, and Kaplan–Meier curves with log-rank tests were plotted for both groupings.

All statistical analyses were performed in R (version 4.6.0; R Foundation for Statistical Computing, Vienna, Austria). A two-sided *p* < 0.05 was considered statistically significant.

## 3. Results

### 3.1. Baseline Characteristics of the Cohort

**Figure A1:**
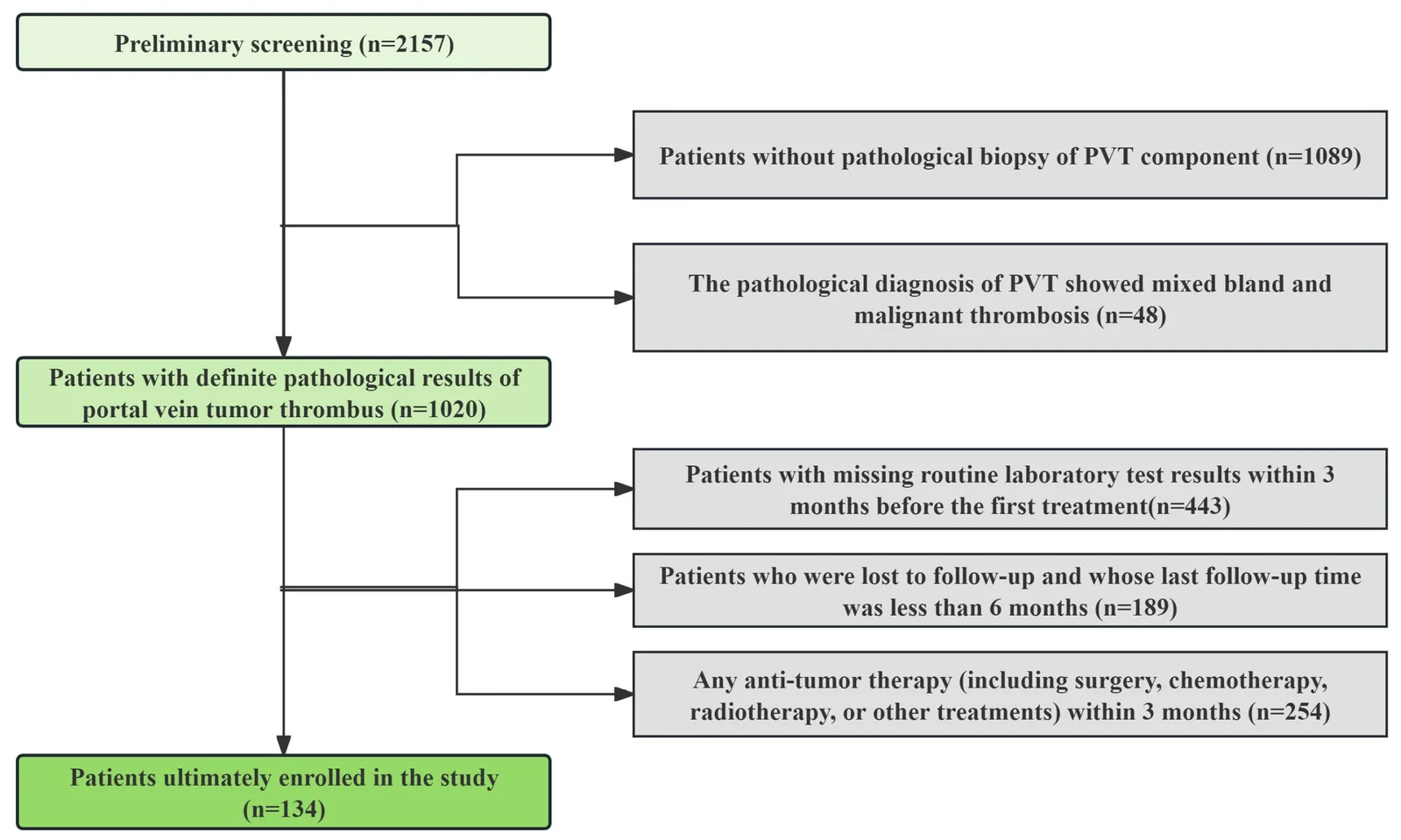
Patient selection flow.

A keyword search of the electronic medical record system initially returned 2157 patients with primary HCC who had undergone contrast-enhanced abdominal CT. After applying the pre-specified criteria, we excluded 1089 patients because they lacked a pathological biopsy of the portal vein thrombus, and another 48 because pathology showed mixed bland and malignant thrombosis. Of the remaining 1020 patients with confirmed PVTT, we further removed 443 who had missing laboratory data within three months before treatment, 189 whose last follow-up was shorter than six months, and 254 who had received anti-tumor therapy in the three months before CT. The final study cohort consisted of 134 patients. The selection flow is shown in Figure A1.

The final cohort included 134 patients, split randomly into a training set (*n* = 94) and a validation set (*n* = 40). Men accounted for 69.4% of the sample, and the mean age sat near 50 years. Imaging examination showed cirrhosis in roughly one-third of patients. We found half of patients were Child–Pugh class B and half were class C. This distribution was expected, given that PVTT seldom occurs with well-preserved liver function. Mean maximum tumor diameter exceeded 10 cm and multiple tumors lesions appeared in nearly 30% of cases.

Clinical test results painted a picture of active tumor biology and impaired hepatic reserve. Alpha-fetoprotein (AFP) and transaminase levels were elevated several times above normal, and both total bilirubin and alkaline phosphatase were raised. Albumin hovered near the lower limit of normal. Coagulation parameters were prolonged, consistent with the cirrhotic background. Renal function and blood counts were largely unremarkable. Metabolic markers like body mass index (BMI), glucose and lipids stayed in the low–normal range. The training and validation sets did not differ meaningfully across baseline variables (all *p* > 0.05). Table 1 provides the full baseline characteristics. The median follow-up times were 59.7 months for the overall cohort, 60.2 months for the training set, and 56.9 months for the validation set. During follow-up, 104 deaths (77.6%) occurred in the overall cohort, with 76 (80.9%) in the training set and 28 (70.0%) in the validation set. The median overall survival was 15.0 months for the entire cohort, 15.1 months for the training set, and 13.9 months for the validation set. Detailed survival event summaries are provided in Table A1.

### 3.2. Clinical Model Construction

Before building the clinical model, we screened 34 candidate variables for collinearity using the variance inflation factor (VIF). Twelve variables exceeded the threshold of 5 and were removed: total cholesterol, triglycerides, prothrombin time, fasting glucose, D-dimer, hemoglobin, activated partial thromboplastin time, albumin, aspartate aminotransferase, alanine aminotransferase, neutrophil percentage, and lymphocyte percentage. The remaining 22 variables had VIF values between 1.36 and 4.97 and were retained for univariate Cox regression. The full VIF results are given in Table A2.

To assess whether the exclusion of highly collinear variables introduced omitted-variable bias, we performed sensitivity analyses by individually forcing key VIF-removed variables (albumin, AST, ALT, PT, and APTT) into the final model alongside the retained predictors. None of these variables was independently associated with survival (all *p* > 0.05), and the C-index remained virtually unchanged across all models (all changes < 0.006). Detailed results are provided in Supplementary Table S1.

After the collinearity screening, 22 variables entered univariate Cox regression. Eleven of them were associated with overall survival at *p* < 0.05: cirrhosis, AFP, BMI, TyG, HDL-C, LDL-C, ALP, total protein, LDH, BUN, and NLR. Age reached the borderline of significance (*p* = 0.050). Among these, higher BMI and higher HDL-C were protective, while the remaining variables were linked to increased mortality risk. Sex, tumor number, tumor size, Child–Pugh class, TBIL, CREA, UA, WBC, RBC, and platelet count showed no significant association with survival. The detailed univariate results are listed in Table 2. A total of 12 variables were carried forward into multivariate analysis.

Twelve variables with *p* < 0.10 in the univariate screening entered the multivariate Cox model. Backward elimination retained four independent predictors: AFP (HR 1.001, 95% CI 1.000–1.002, *p* = 0.003), BMI (HR 0.874, 95% CI 0.771–0.990, *p* = 0.034), HDL-C (HR 0.099, 95% CI 0.013–0.725, *p* = 0.023), and ALP (HR 1.002, 95% CI 1.000–1.004, *p* = 0.017). Higher AFP and ALP signaled worse survival, whereas higher BMI and HDL-C were protective. Age, TyG, LDL-C, total protein, LDH, BUN, NLR, and cirrhosis were removed during backward elimination. Table 3 presents the full model and the final model side by side.

### 3.3. Radiomics Feature Selection and Rad-Score Construction

**Figure 1:**
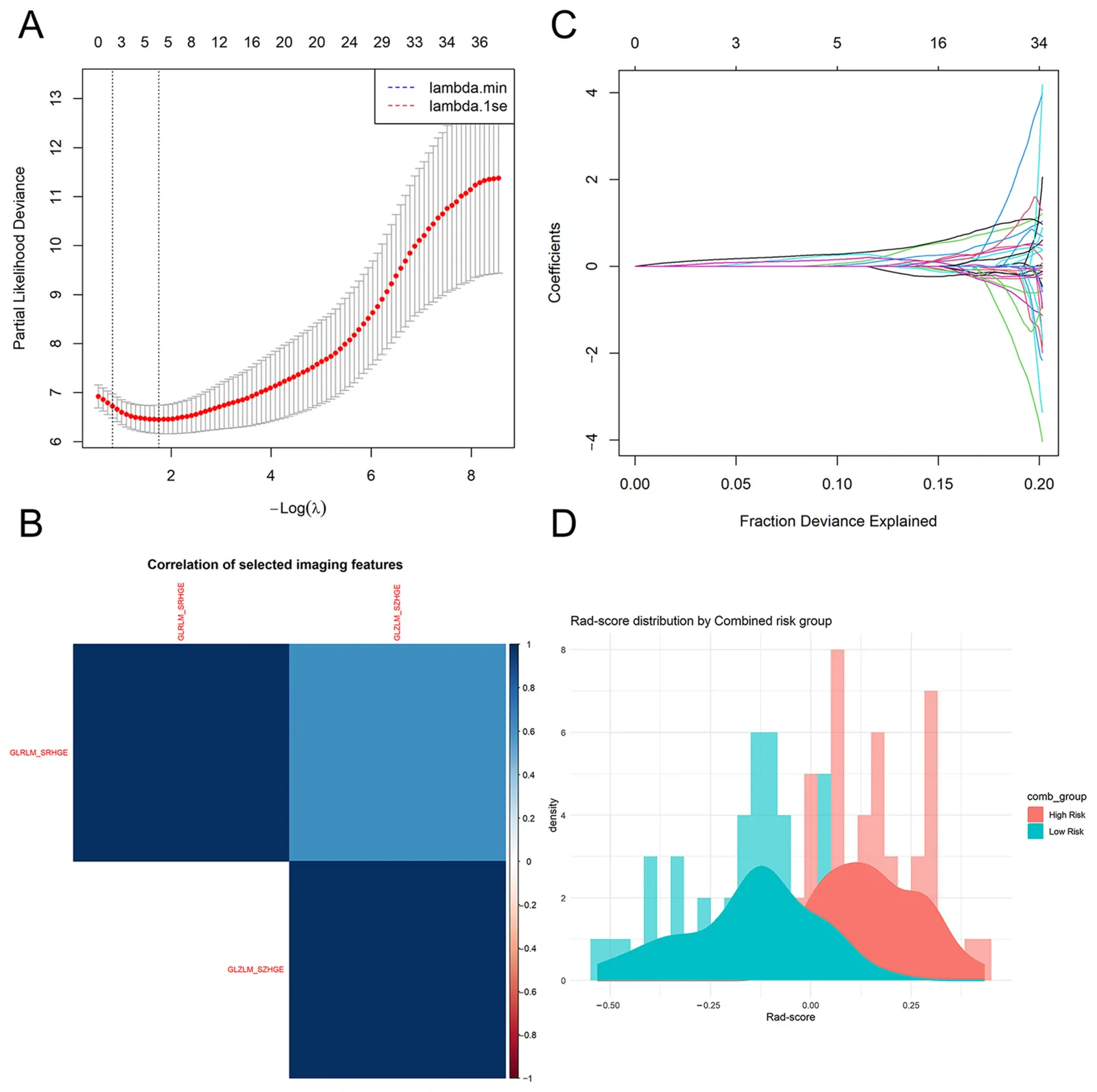
Radiomics feature selection and Rad-score construction. (**A**) LASSO cross-validation curve. The left dashed line marks λ.min, and the right dashed line marks λ.1se. Two features were retained at λ.1se. (**B**) Coefficient profile plot. Each colored line traces the coefficient of one radiomic feature as the penalty λ changes. Only two features survived at the selected λ. (**C**) Correlation heatmap of the two selected features. (**D**) Distribution of the Rad-score in the training set, stratified by the combined-model risk group (high-risk vs. low-risk).

A total of 43 radiomic features were extracted from the portal venous phase CT images and Z-score normalized using the training set statistics. The LASSO algorithm was then applied with 10-fold cross-validation. The cross-validation procedure yielded λ.min = 0.173 and λ.1se = 0.439, with the full regularization path ranging from 0.0002 to 0.580; the more conservative λ.1se was selected for the final model to prioritize generalizability. Two features with non-zero coefficients at λ.1se were retained: GLRLM_SRHGE (coefficient 0.078265) and GLZLM_SZHGE (coefficient 0.154322). The Rad-score was built as their linear combination: Rad-score = 0.078265 × GLRLM_SRHGE + 0.154322 × GLZLM_SZHGE. The same normalization parameters and formula were applied to the validation set without modification (Figure 1).

To assess the stability of this radiomic signature, we performed 1000 bootstrap resamples of the training set using the same λ.min = 0.173. The two originally selected features—GLRLM_SRHGE and GLZLM_SZHGE—were re-selected in 61.7% and 26.8% of the bootstrap iterations, respectively, with an average of 2.4 features selected per iteration. When grouping features by radiomic family, at least one high-gray-level texture feature (from the GLRLM or GLZLM families) was selected in 84.6% of the bootstraps, indicating that the model consistently captures the prognostic signal of tumor heterogeneity, although the exact surrogate feature may vary depending on the sampling.

### 3.4. Model Development and Formulas

We built three Cox proportional hazards models from the training set. The clinical model contained the four independent predictors that backward elimination retained: AFP, BMI, HDL-C, and ALP. The Rad-score model relied solely on the Rad-score, which combines the two radiomic features kept by LASSO. For the combined model, we integrated all four clinical variables and the Rad-score into a single formula. Table 4 lists the three formulas. All continuous variables enter the formulas in their original measured units.

**Figure 2:**
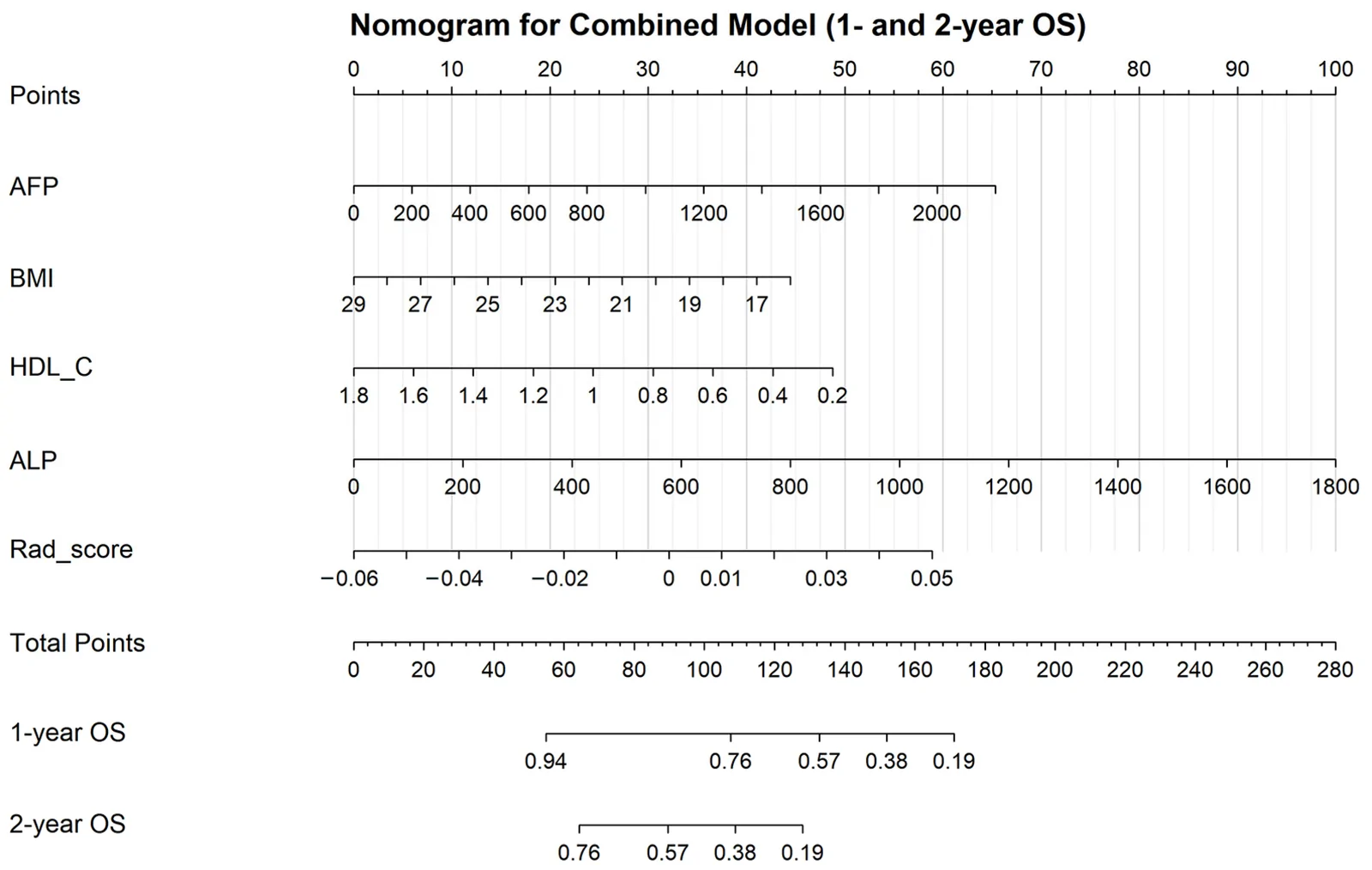
Nomogram for the combined model predicting 1-year and 2-year overall survival in hepatocellular carcinoma (HCC) patients with portal vein tumor thrombus (PVTT).

We built a nomogram from the combined model to translate the prognostic information into a practical tool. The nomogram incorporated all five variables that entered the combined model: AFP, BMI, HDL-C, ALP, and the Rad-score, and returned predicted survival probabilities at 12 and 24 months. For each patient, the total points were calculated by summing the individual contributions of the five predictors, and the corresponding 1-year and 2-year overall survival probabilities were read from the bottom scales. Figure 2 displays the nomogram.

### 3.5. Model Performance and Discrimination

We evaluated the discrimination of the three models in both the training and validation sets using the C-index and time-dependent AUC at 12 and 24 months. The combined model gave the highest C-index in the training set (0.843, 95% CI 0.769–0.917), followed by the clinical model (0.831) and the Rad-score (0.785). The same ranking held in the validation set, where the combined model reached a C-index of 0.815 (95% CI 0.737–0.893), the clinical model 0.808, and the Rad-score 0.755.

**Figure 3:**
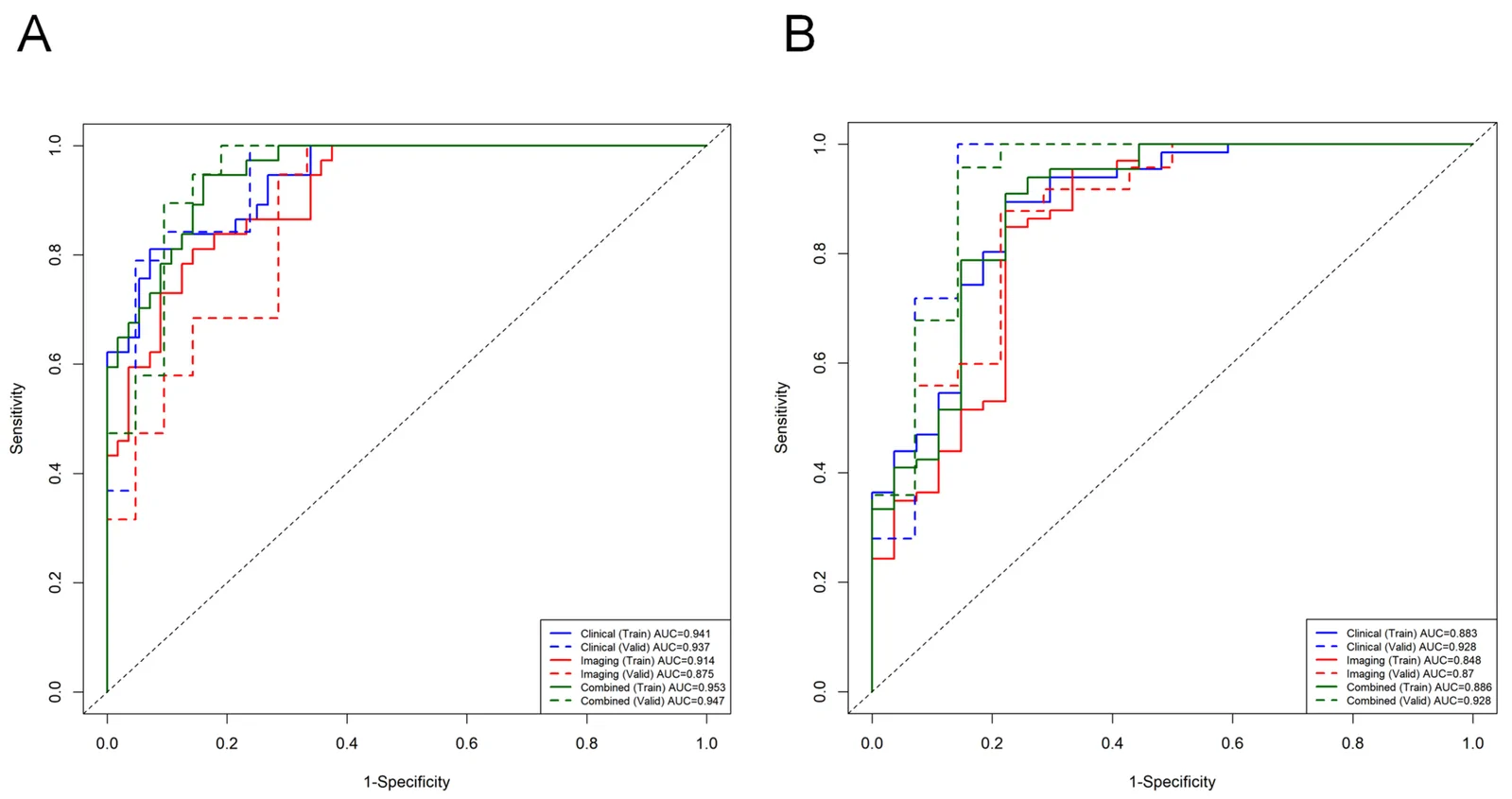
ROC curves for predicting 1-year and 2-year overall survival. (**A**) 1-year ROC curves for the training set (solid lines) and validation set (dashed lines). (**B**) 2-year ROC curves for the training set (solid lines) and validation set (dashed lines).

For 1-year AUC, the combined model outperformed the other two in both datasets. In the training set, its 1-year AUC was 0.953, compared with 0.941 for the clinical model and 0.914 for the Rad-score. The advantage persisted in the validation set: 0.947 for combined, 0.937 for clinical model, and 0.875 for Rad-score. At 2 years, the combined and clinical models had similar AUC values in the validation set (both 0.928), whereas the Rad-score dropped to 0.870. In the training set, the 2-year AUC reached 0.886 for the combined model, 0.883 for the clinical model, and 0.848 for the Rad-score alone. The ROC curves at both time points are given in Figure 3, with training and validation sets overlaid. Across the board, the combined model led on discrimination. The Rad-score by itself sat at the bottom, though adding it to the clinical predictors pushed both the C-index and the AUC upward. Table 5 lists the full set of performance metrics.

To further evaluate the robustness of the combined model against overfitting, we performed bootstrap optimism correction with 200 resamples of the training set. The apparent C-index of 0.843 was corrected to 0.830, with an optimism of only 0.013, indicating that the model’s discriminative performance is not unduly driven by the specific composition of the training cohort and is likely to generalize to similar patient populations.

We tested the proportional hazards assumption for the final combined model using Schoenfeld residual analysis. AFP (*p* = 0.094), BMI (*p* = 0.240), and HDL-C (*p* = 0.213) satisfied the proportional hazards assumption, while ALP (*p* = 0.00052) and Rad-score (*p* = 0.00323) showed time-varying effects. The global test was also significant (*p* = 0.00643). Detailed results are provided in Table A3.

**Figure 4:**
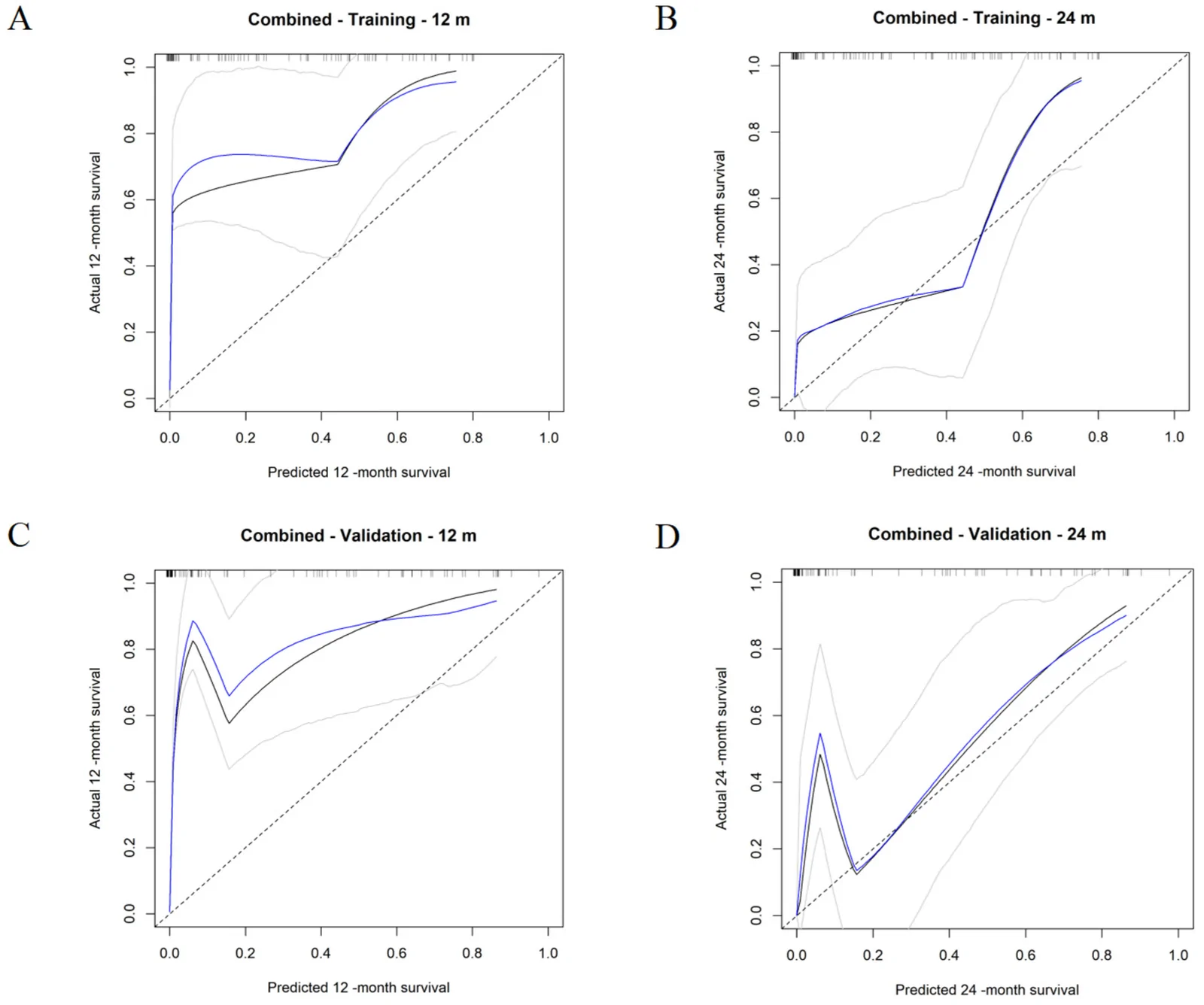
Calibration curves of the combined model for predicting 1-year and 2-year overall survival. (**A**) Training set, 12 months. (**B**) Training set, 24 months. (**C**) Validation set, 12 months. (**D**) Validation set, 24 months. The dashed diagonal line represents perfect calibration. The solid line shows the actual calibration performance. Points indicate observed survival probabilities within each risk decile, with error bars representing 95% confidence intervals obtained from bootstrap resampling.

We also checked calibration. Calibration curves for the combined model were generated at 12 and 24 months in the training set and internal validation set. The calibration intercepts ranged from 1.030 to 1.222, indicating that the model systematically underestimates survival probabilities, particularly in the validation set. The slopes ranged from 1.044 to 1.291, indicating overdispersion—predictions are too extreme, with high-risk patients estimated as sicker and low-risk patients as healthier than their true risk. This pattern does not compromise the model’s rank-ordering ability (as reflected by the C-index and AUC), but it suggests that external recalibration may be needed before the model is used to generate absolute survival probability estimates in clinical practice. The slope and intercept values are tabulated in Table A4 (Figure 4).

### 3.6. Risk Stratification and Survival Analysis

We dichotomized patients into high-risk and low-risk groups using the median risk score. The optimal cut-off derived from Youden’s index gave unbalanced splits in some settings and was therefore set aside. The median threshold produced usable comparisons for all three models in both the training and validation sets.

**Figure 5:**
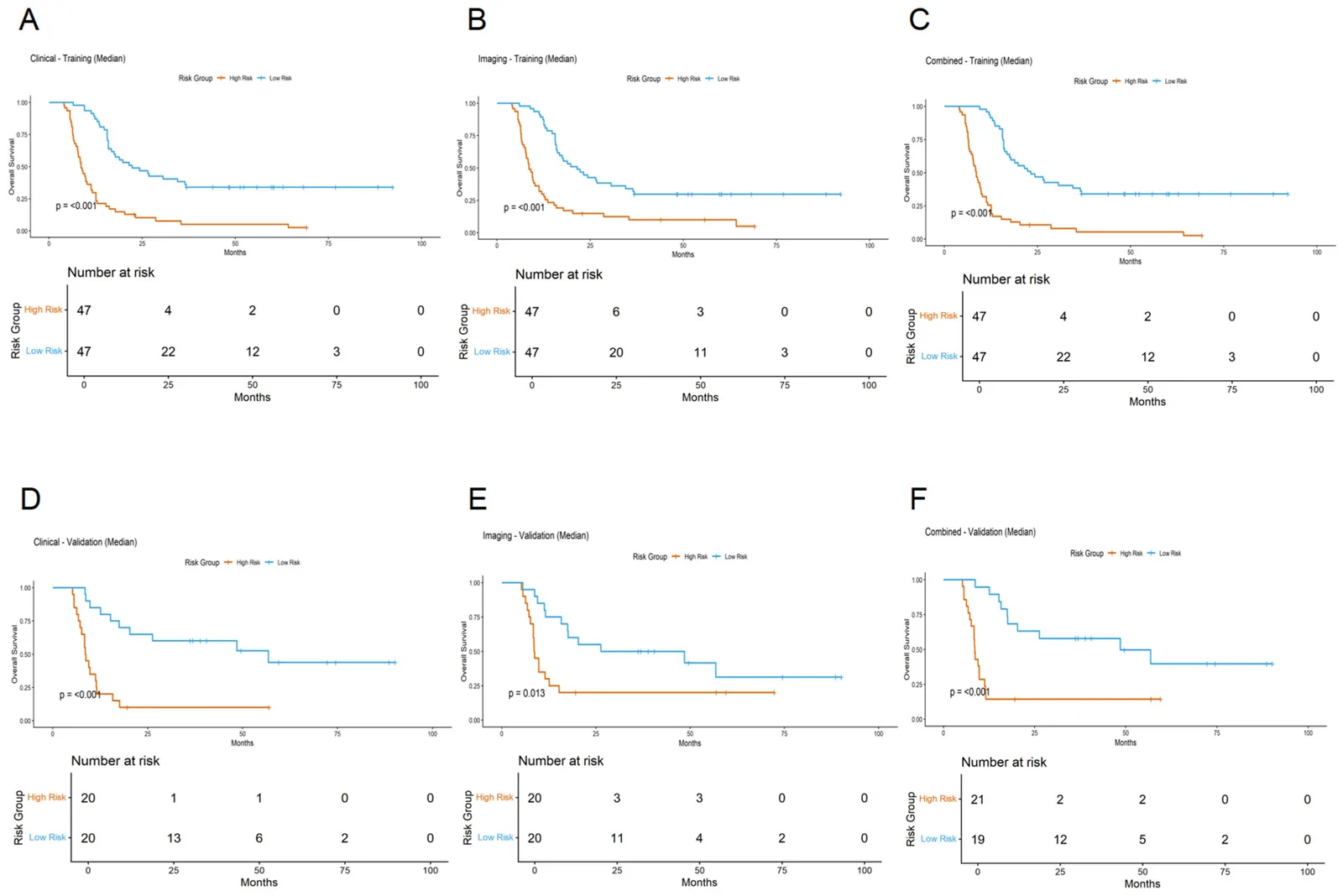
Kaplan–Meier survival curves for the three models stratified by the median risk score. (**A**–**C**) Training set: (**A**) clinical model, (**B**) imaging model, (**C**) combined model. (**D**–**F**) Validation set: (**D**) clinical model, (**E**) imaging model, (**F**) combined model. High-risk and low-risk groups were defined by the median risk score in each dataset. *p* values were obtained from the log-rank test. The risk table below each panel shows the number of patients at risk at each time point.

In the training set, the three models all separated the survival curves sharply (log-rank *p* < 0.001). The combined model spread the curves the furthest apart, with the low-risk group keeping a 12-month survival above 0.80 and the high-risk group falling fast. The clinical model gave a similar pattern, and the imaging model still produced a clear split, though the gap was narrower. The validation set told roughly the same story. Both the combined and clinical models maintained their high–low separation (*p* < 0.001). The imaging model also reached significance, though with a slightly larger *p* value of 0.013. Across both datasets, the combined model yielded the widest survival divergence, followed by the clinical model and then the imaging model. Figure 5 displays the Kaplan–Meier curves.

**Figure 6:**
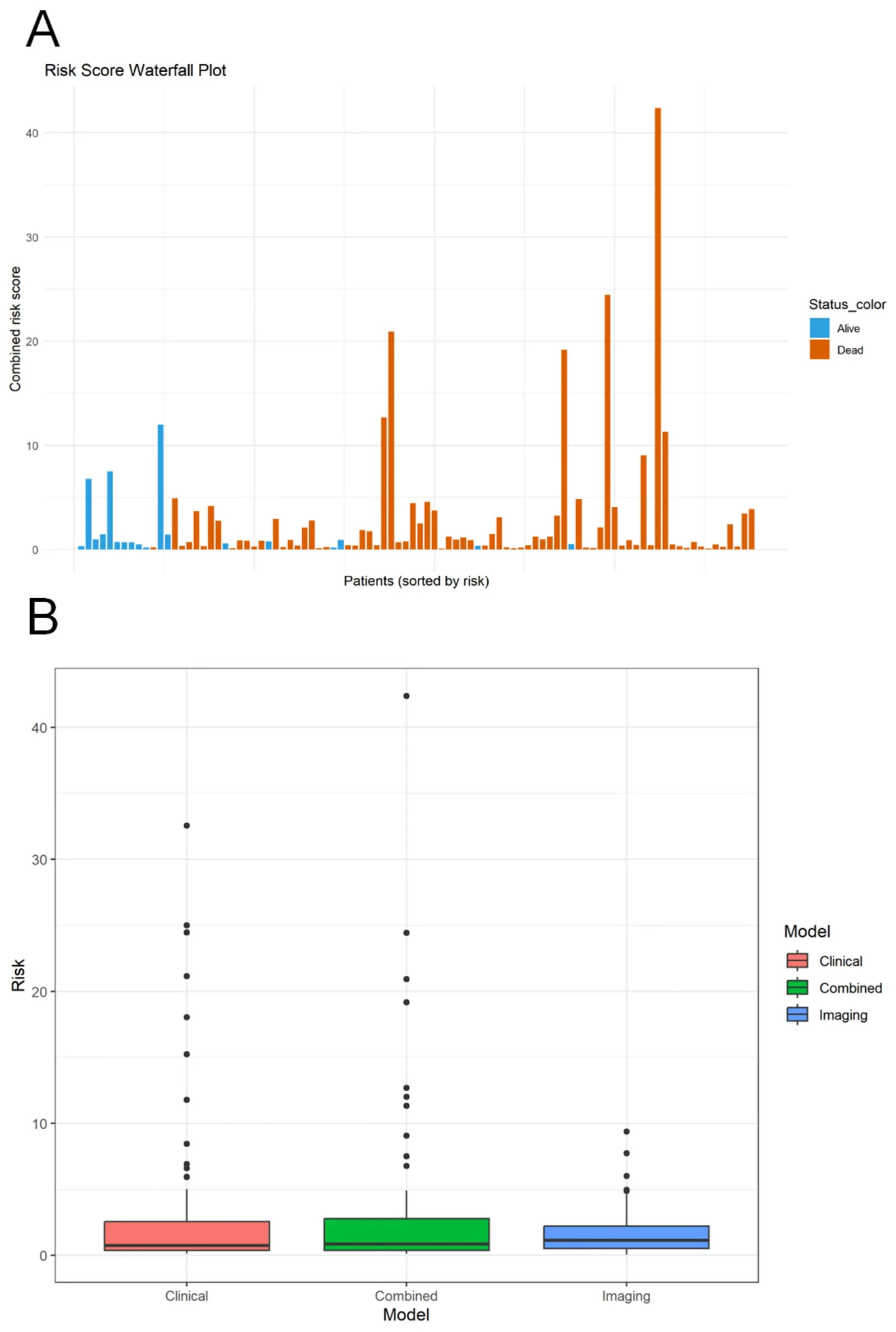
Risk score distribution in the training set. (**A**) Waterfall plot of individual risk scores from the combined model. (**B**) Box plots of risk scores from the clinical, imaging, and combined models.

We ranked patients by their combined-model risk score and displayed them as a waterfall plot (Figure 6A). Deaths clustered heavily on the high-score side, while the low-score side was mostly populated by survivors. We then compared the risk score distributions of the three models using box plots (Figure 6B). The combined model spread the scores the widest, separating high-risk and low-risk patients more clearly than the clinical or imaging models alone.

**Figure 7:**
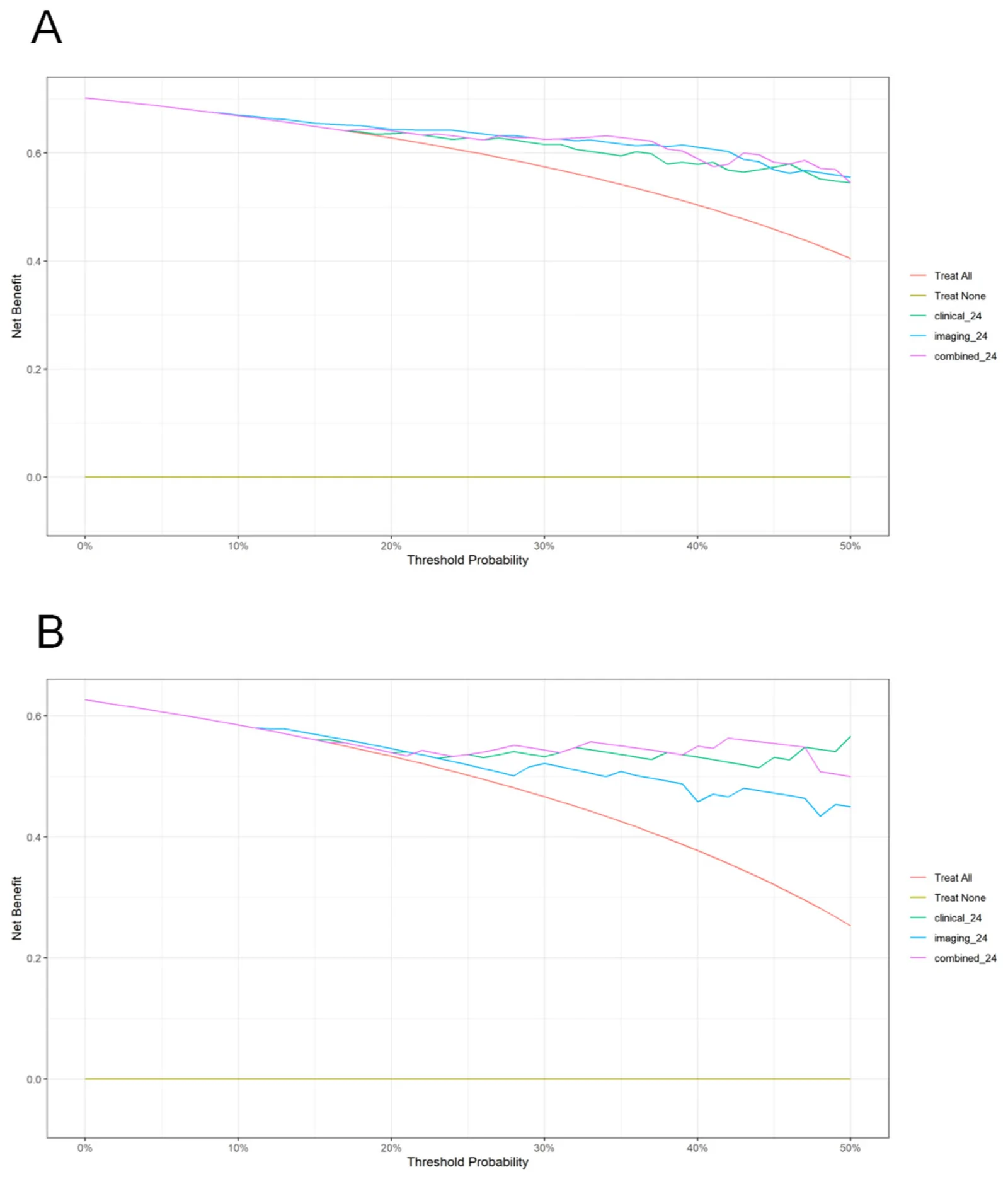
Decision curve analysis of the three models for predicting 2-year overall survival. (**A**) Training set: the combined model provided the highest net benefit across 0–50% thresholds, followed by the clinical model and then the imaging model; all three models exceeded the “treat all” and “treat none” strategies. (**B**) Validation set: the combined model again showed the greatest net benefit, with the same ranking among models, and all remained above the reference strategies.

We ran decision curve analysis at 24 months, first in the training set, then in the validation set. In both datasets, the combined model gave the highest net benefit across most of the 0 to 50 percent threshold range. The clinical model came next, and the imaging model third. All three models remained above the two reference strategies, treat all and treat none, across the clinically relevant thresholds. Figure 7 presents the DCA curves.

## 4. Discussion

We compared three survival models in HCC patients with PVTT, and the combined model came out ahead on discrimination, calibration, and net benefit. Four clinical variables and a radiomics score built from two CT texture features were included in the final model. Routine lab values and AI-pulled imaging features thus appear to carry complementary prognostic weight in this setting.

PVTT is a turning point. It speeds up intrahepatic spread, worsens portal hypertension, and cuts short the window for effective therapy [31,32]. Even with checkpoint inhibitors added to the arsenal, survival above 20 months is uncommon. Still, the “advanced” label hides considerable heterogeneity. Some patients live far longer than the median. Tools that can separate them out, especially tools built from data that are already available, could help clinicians decide how aggressively to treat without piling on extra cost. Before discussing the model’s performance, it is important to acknowledge several constraints related to patient selection. The requirement for pathological confirmation of PVTT excluded a substantial proportion of patients (1089/2157, 50.5%) and may have selected a healthier, more procedure-tolerant subgroup, limiting generalizability. While this choice ensured diagnostic accuracy—distinguishing tumor thrombus from bland thrombus—it does not reflect routine clinical practice where imaging-based diagnosis is standard. Similarly, we excluded 189 patients with follow-up shorter than six months and 254 who had received recent anti-tumor therapy (including TACE, radiotherapy, targeted therapy, immunotherapy, or local ablation) within three months prior to CT. These exclusions were intended to ensure that baseline data reflected the untreated tumor state and to maintain completeness of follow-up data. However, combined with the pathological confirmation requirement, these criteria narrowed the cohort to a highly selected population (134/2157, 6.2%), which may limit the generalizability of our findings. We also acknowledge that a formal comparison of baseline characteristics between included and excluded patients could not be performed. A substantial proportion of excluded patients had incomplete baseline data at the time of their initial visit—many were lost to follow-up or transferred to other institutions before completing the recommended evaluations—which was one of the reasons for their exclusion. Thus, we cannot formally assess whether the analytic cohort is fully representative of the entire screened PVTT population. We also acknowledge that only 35.1% of patients had documented evidence of cirrhosis, whereas all patients were Child–Pugh B or C. Cirrhosis was recorded based on any available clinical evidence (history, imaging, or clinical assessment). However, all patients underwent contrast-enhanced CT as part of the study inclusion criteria; the 35.1% figure reflects those with clearly documented cirrhosis in the medical records, not the prevalence of cirrhosis. Hepatic dysfunction in this cohort may therefore reflect a combination of chronic liver disease, tumor burden, and PVTT-related portal hypertension, rather than cirrhosis alone. These factors should be considered when interpreting the model’s performance.

The clinical predictors that survived backward elimination all have a plausible biological footing. AFP is the oldest biomarker in HCC, and its independent link to survival held in our cohort [33,34,35]. BMI and HDL-C were both protective. A higher BMI in advanced HCC is unlikely to reflect classic obesity-related risk; it more likely signals preserved lean mass and nutritional buffer that help patients withstand the metabolic drain of a large tumor. This aligns with the “obesity paradox” noted by several groups [36,37,38]. HDL-C may point in a similar direction. Low HDL-C has been linked to systemic inflammation and cancer cachexia. In our data, each increment in HDL-C was tied to a sizable drop in mortality, consistent with reports that lipid handling is deeply altered in advanced liver cancer [39,40]. Alkaline phosphatase, the fourth predictor, could flag biliary obstruction, heavy hepatic infiltration, or bone involvement, all of which worsen the outlook [41].

The radiomics part, though pared down to two features, still picked up a prognostic signal that the blood variables missed. GLRLM_SRHGE and GLZLM_SZHGE belong to the high-gray-level run-length and zone-size families. Their positive coefficients mean that tumors with coarser texture and more frequent high-intensity runs on CT foreshadow higher mortality. Other groups have reported that similar texture features carry prognostic value across several cancers, HCC included [42,43,44]. Biologically, these features may encode necrosis, hemorrhage, or architectural disarray that routine radiology does not formally grade [45,46,47]. A practical point worth underlining: the radiomics score came from a single portal-venous phase CT, a scan already embedded in the standard workup for PVTT. No extra imaging, no additional contrast, no biopsy. That makes the approach compatible with everyday clinical workflow rather than a research-only exercise. Several technical limitations of the radiomic analysis should be noted. Formal reproducibility metrics for ROI delineation (such as ICC or Dice coefficients) were not calculated because independent duplicate segmentations were not preserved, although contours were reviewed by two experienced radiologists with consensus resolution. Detailed CT acquisition and reconstruction parameters are provided in Supplementary Material 1; all scans were performed using the same scanner model following the center’s standard protocol, so no scanner-specific harmonization (e.g., ComBat) was required. Furthermore, the exact identity of individual features selected by LASSO may be sensitive to sampling variability—using 1000 bootstrap resamples with λ.min, the two retained features showed re-selection frequencies of 61.7% and 26.8%—though the composite Rad-score remained robust (optimism-corrected C-index = 0.830). Finally, radiomics feature interpretation, while tied to texture theory, remains inferential without histopathological correlation.

Our combined model yielded a C-index of 0.843 in the training set and 0.815 in the validation set, with 1-year AUCs above 0.94. These numbers sit in the same range as—and sometimes above—published models that depend on genomic panels, circulating tumor DNA, or advanced MRI techniques. Those tools are powerful but are also expensive and geographically concentrated. Our predictors come from a basic blood draw and a single contrast-enhanced CT. The cost difference matters: HCC is most prevalent in regions where healthcare budgets are thin, and a model that runs on what is already collected can reach far more patients. Calibration assessment showed slopes ranging from 1.044 to 1.291 and intercepts from 1.030 to 1.222. These values indicate a tendency toward mild overdispersion, particularly in the validation set. While this suggests that the nomogram’s absolute probability estimates should be interpreted with caution and external recalibration is recommended, the model maintained strong rank-ordering ability (as reflected by the C-index and AUC) and demonstrated net benefit on decision curve analysis. The decision curves reinforced this picture: across thresholds up to 50%, the combined model net benefit stayed above the single-domain models, and all three models beat the treat-all and treat-none strategies.

Two external benchmarks help place our results in context. A multicenter study of 1026 HBV-related HCC patients without PVTT identified male sex, low albumin-to-alkaline-phosphatase ratio, elevated aspartate-to-platelet ratio, extrahepatic metastasis, and multiple tumors as risk factors for PVTT occurrence, and generated nomograms for PFS and OS with C-indexes around 0.72–0.80 [48]. That study asked who will develop PVTT. Our study starts where that question ends, focusing on patients who already have the thrombus. In this population, our combined model reached C-indices of 0.843 (training) and 0.815 (validation), which are descriptively higher than their OS nomogram. Another study, also in HCC patients with PVTT, built a radiomics-based nomogram that included clinical and radiotherapy dosimetric variables for predicting OS after radiation, with a C-index of 0.73 [49]. Our model also showed descriptively higher C-indices and did not require dosimetric data, so it can be applied before treatment selection rather than being tied to a specific modality. It should be noted, however, that these comparisons are descriptive only; formal statistical testing could not be performed because individual patient data from the external studies were not accessible. We acknowledge that the discriminative performance observed in our study—particularly the C-index of 0.843 in the training set and 0.815 in the validation set—may be partly optimistic. Several factors may contribute to this. First, the stringent inclusion criteria (pathological confirmation, exclusion of recent therapy, and complete follow-up) selected a relatively homogeneous patient subset, which can inflate performance relative to unselected real-world populations. Second, the validation set was derived from a random split of the same single-center cohort rather than an external dataset, which may underestimate performance degradation in independent settings. Third, the moderate sample size (*n* = 94 for training) increases the risk of overfitting, despite the use of LASSO regularization and bootstrap optimism-correction. Finally, the multi-step modeling process—involving VIF screening, univariate selection, backward elimination, and LASSO—may have capitalized on chance associations specific to this cohort. To mitigate overfitting, we applied LASSO with 10-fold cross-validation and reported optimism-corrected C-index (0.830), which remains robust. Nevertheless, cautious interpretation is warranted, and external validation in larger, multicenter cohorts is essential before clinical implementation. Taken together, the two external comparisons suggest that blending routine laboratory values with CT texture features may sharpen survival prediction in this high-risk group, although head-to-head validation in a shared dataset is needed to confirm this observation.

Several additional limitations merit consideration. The study was retrospective and single-center, and the internal validation set was derived from the same cohort by random split, which cannot demonstrate external generalizability. The sample size was modest (*n* = 94 for training), and the model’s calibration showed a tendency toward overdispersion (slopes up to 1.291), suggesting that absolute survival probability estimates should be interpreted with caution and external recalibration is warranted. The positive intercepts (1.030–1.222) indicate that the model systematically underestimates survival probabilities, further supporting the need for caution when interpreting absolute survival estimates. Tumor thrombus extent was not graded by a formal system. Schoenfeld residual analysis detected time-varying effects for ALP and Rad-score. This is clinically plausible: ALP levels may fluctuate with biliary obstruction or bone involvement during disease progression, while Rad-score—derived from baseline CT—primarily reflects initial tumor heterogeneity and necrosis, which exert the strongest prognostic influence during the early post-treatment period. As time elapses, subsequent therapies and disease progression increasingly shape long-term survival. This pattern does not compromise the model’s intended use as a baseline risk stratification tool, as the strongest predictive signal occurs during the critical early treatment decision window. However, caution is warranted when extrapolating predictions to very long-term follow-up. Importantly, we did not systematically capture post-enrollment anticancer treatments or concomitant medications, which may have influenced long-term survival and metabolic markers. Despite these limitations, the model maintained strong discriminative performance and net benefit on decision curve analysis. Finally, multicenter prospective studies with external CT datasets are the logical next step to validate and refine these findings.

## 5. Conclusions

We tested three prognostic models in HCC patients with PVTT. The combined model, which integrates four routine clinical variables and a CT-based radiomics score, outperformed the clinical and imaging models alone. The nomogram derived from this model provides individualized 12- and 24-month survival estimates using inputs from a basic blood panel and a single portal-venous phase CT. This internally validated model represents a pragmatic first step toward risk-stratified treatment decisions in this population. However, prospective, multicenter, external validation is essential before the nomogram can be considered for routine clinical implementation.

## Figures and Tables

**Table 1:** Baseline characteristics of all patients.

Characteristic	Overall (*n* = 134)	Training Set (*n* = 94)	Validation Set (*n* = 40)	*p*
Sex, *n*				0.366
Male	93 (69.4%)	63 (67.0%)	30 (75.0%)	
Female	41 (30.6%)	31 (33.0%)	10 (25.0%)	
Age (y), mean ± SD	50.5 ± 9.7	51.6 ± 9.1	50.1 ± 11.1	0.441
Liver cirrhosis, *n* (%)				0.545
Yes	47 (35.1%)	35 (37.2%)	12 (30.0%)	
No	87 (64.9%)	59 (62.8%)	28 (70.0%)	
Child–Pugh, *n* (%)				0.273
B	65 (48.5%)	49 (52.1%)	16 (40.0%)	
C	69 (51.5%)	45 (47.9%)	24 (60.0%)	
Maximum tumor diameter (cm), mean ± SD	10.1 ± 4.1	10.4 ± 4.2	9.4 ± 3.7	0.367
Number of tumors, *n* (%)				0.440
Single	95 (70.9%)	69 (73.4%)	26 (65.0%)	
Multiple	39 (29.1%)	25 (26.6%)	14 (35.0%)	
AFP (ng/mL), mean ± SD	749.6 ± 540.4	785.2 ± 554.2	672.8 ± 493.7	0.249
ALT (U/L), mean ± SD	119.9 ± 140.4	128.6 ± 139.5	116.3 ± 133.7	0.453
AST (U/L), mean ± SD	190.0 ± 211.2	192.1 ± 212.3	171.6 ± 200.1	0.475
ALP (U/L), mean ± SD	469.9 ± 299.7	479.2 ± 320.9	434.6 ± 226.1	0.829
TBIL (μmol/L), mean ± SD	149.9 ± 78.1	158.5 ± 85.2	140.0 ± 57.1	0.335
TP (g/L), mean ± SD	69.7 ± 10.9	69.8 ± 10.9	69.5 ± 11.1	0.963
ALB (g/L), mean ± SD	38.8 ± 9.2	38.8 ± 9.1	38.8 ± 9.5	0.996
PT (s), mean ± SD	15.2 ± 3.3	15.12 ± 3.5	15.4 ± 2.9	0.300
APTT (s), mean ± SD	38.2 ± 9.7	38.0 ± 10.3	38.5 ± 8.4	0.225
D-dimer (mg/L), mean ± SD	2.4 ± 2.1	2.4 ± 2.3	2.3 ± 1.6	0.461
WBC (×10^9^/L), mean ± SD	6.4 ± 2.2	6.4 ± 2.4	6.4 ± 1.7	0.985
NEUT (%), mean ± SD	61.5 ± 10.2	61.9 ± 10.9	60.7 ± 8.3	0.547
LYM (%), mean ± SD	27.7 ± 8.6	27.4 ± 9.0	28.5 ± 7.7	0.442
NLR, mean ± SD	2.9 ± 2.5	2.95 ± 2.6	2.7 ± 2.3	0.523
RBC (×10^12^/L), mean ± SD	4.4 ± 0.8	4.4 ± 0.9	4.3 ± 0.6	0.438
Hb (g/L), mean ± SD	129.5 ± 20.9	126.2 ± 21.7	127.5 ± 19.2	0.710
PLT (×10^9^/L), mean ± SD	180.4 ± 60.8	179.1 ± 64.3	173.3 ± 52.1	0.366
CREA (μmol/L), mean ± SD	83.9 ± 16.8	84.9 ± 17.9	81.4 ± 13.8	0.306
BUN (mmol/L), mean ± SD	6.6 ± 2.3	6.8 ± 2.5	6.2 ± 1.9	0.603
UA (μmol/L), mean ± SD	349.9 ± 56.7	348.5 ± 59.7	343.3 ± 49.4	0.198
Plasma glucose (mmol/L), mean ± SD	7.5 ± 2.1	7.54 ± 2.1	7.6 ± 2.3	0.898
BMI, mean ± SD	22.0 ± 2.7	22.1 ± 2.8	22.0 ± 2.6	0.836
TC (mmol/L), mean ± SD	4.3 ± 1.3	4.3 ± 1.3	4.4 ± 1.5	0.845
TG (mmol/L), mean ± SD	2.7 ± 1.3	2.7 ± 1.3	2.8 ± 1.5	0.926
TyG index, mean ± SD	9.6 ± 0.9	9.6 ± 0.8	9.5 ± 1.0	0.915
HDL-C (mmol/L), mean ± SD	1.0 ± 0.3	1.0 ± 0.3	1.0 ± 0.4	0.683
LDL-C (mmol/L), mean ± SD	2.4 ± 0.9	2.4 ± 0.9	2.4 ± 1.0	0.512
LDH (U/L), mean ± SD	420.2 ± 250.1	424.2 ± 252.1	424.3 ± 230.7	0.806

Abbreviations: AFP, alpha-fetoprotein; ALB, albumin; ALP, alkaline phosphatase; ALT, alanine aminotransferase; APTT, activated partial thromboplastin time; AST, aspartate aminotransferase; BMI, body mass index; BUN, blood urea nitrogen; CREA, creatinine; Hb, hemoglobin; HDL-C, high-density lipoprotein cholesterol; LDH, lactate dehydrogenase; LDL-C, low-density lipoprotein cholesterol; LYM, lymphocyte percentage; NEUT, neutrophil percentage; NLR, neutrophil-to-lymphocyte ratio; PLT, platelet; PT, prothrombin time; RBC, red blood cell count; TBIL, total bilirubin; TC, total cholesterol; TG, triglycerides; TyG, triglyceride-glucose index; UA, uric acid; WBC, white blood cell count.

**Table 2:** Univariate logistic regression analyses of the clinical model.

Variable	Hazard Ratio	95% Confidence Interval	*p*
Age	1.025	1.000–1.051	0.050
Sex	0.988	0.609–1.603	0.960
Number of tumors	1.138	0.693–1.870	0.609
Tumor size	0.981	0.929–1.036	0.491
Cirrhosis	1.648	1.036–2.620	0.035
Child–Pugh	1.155	0.736–1.811	0.531
AFP	1.001	1.001–1.001	<0.001
BMI	0.702	0.627–0.786	<0.001
TyG index	2.465	1.890–3.216	<0.001
HDL-C	0.117	0.056–0.245	<0.001
LDL-C	0.661	0.472–0.926	0.016
TBIL	0.999	0.996–1.002	0.593
ALP	1.003	1.003–1.004	<0.001
Total protein	0.969	0.943–0.996	0.025
LDH	1.003	1.002–1.004	<0.001
CREA	1.012	0.998–1.026	0.102
UA	1.002	0.998–1.007	0.246
BUN	1.151	1.047–1.264	0.003
WBC	0.942	0.843–1.054	0.298
NLR	1.149	1.062–1.242	0.001
RBC	0.955	0.694–1.315	0.778
Platelet	0.997	0.993–1.001	0.156

**Table 3:** Multivariate Cox regression: independent predictors of overall survival.

Variable	Hazard Ratio	95% Confidence Interval	*p*
Age	1.004	0.972–1.037	0.820
AFP	1.001	1–1.002	0.003
BMI	0.874	0.771–0.99	0.034
TyG	0.725	0.323–1.629	0.436
HDL-C	0.099	0.013–0.725	0.023
LDL-C	0.607	0.291–1.267	0.183
ALP	1.002	1–1.004	0.017
Total-protein	1.061	0.989–1.138	0.098
LDH	0.999	0.997–1.002	0.609
BUN	1.013	0.873–1.175	0.863
NLR	1.026	0.903–1.166	0.695
Cirrhosis			
Yes	0.764	0.295–1.979	0.580
No	ref	ref	/

**Table 4:** Linear predictor formulas for risk scores.

Model	Risk Score Formula
Clinical	Clinical score = 0.0011 * AFP − 0.1081 * BMI − 1.4369 * HDL-C + 0.0016 * ALP
Rad-score	Rad-score = 0.0783 * GLRLM_SRHGE + 0.1543 * GLZLM_SZHGE
Combined	Combined score = 0.0011 * AFP − 0.1081 * BMI − 1.4369 * HDL-C + 0.0016 * ALP + 0.0783 * GLRLM_SRHGE + 0.1543 * GLZLM_SZHGE

**Table 5:** Discrimination performance of the three models in the training and validation sets.

Dataset/Model	Time	C-Index	C-Index 95% CI	AUC
Training set				
Clinical	1-year	0.831	0.755–0.907	0.941
Clinical	2-year	0.831	0.755–0.907	0.883
Imaging	1-year	0.785	0.702–0.868	0.914
Imaging	2-year	0.785	0.702–0.868	0.848
Combined	1-year	0.843	0.769–0.917	0.953
Combined	2-year	0.843	0.769–0.917	0.886
Validation set				
Clinical	1-year	0.808	0.728–0.888	0.937
Clinical	2-year	0.808	0.728–0.888	0.928
Imaging	1-year	0.755	0.668–0.842	0.875
Imaging	2-year	0.755	0.668–0.842	0.870
Combined	1-year	0.815	0.737–0.893	0.947
Combined	2-year	0.815	0.737–0.893	0.928

## Data Availability

Due to ethical restrictions protecting patient privacy and institutional policies requiring formal approval for data sharing, individual patient data cannot be shared publicly. De-identified data is available from the corresponding author upon reasonable request and subject to institutional approval.

## Supplementary Materials

The following supporting information can be downloaded at: https://www.mdpi.com/article/10.3390/biomedicines14091894/s1, Supplementary Table S1: Sensitivity analyses for VIF-removed variables.

## Abbreviations

The following abbreviations are used in this manuscript:

AFP	alpha-fetoprotein
ALP	alkaline phosphatase
ALT	alanine aminotransferase
APTT	activated partial thromboplastin time
AST	aspartate aminotransferase
BMI	body mass index
BUN	blood urea nitrogen
CREA	creatinine
Hb	hemoglobin
HDL-C	high-density lipoprotein cholesterol
LDH	lactate dehydrogenase
LDL-C	low-density lipoprotein cholesterol
LYM	lymphocyte percentage
NEUT	neutrophil percentage
NLR	neutrophil-to-lymphocyte ratio
PLT	platelet
PT	prothrombin time
RBC	red blood cell count
TBIL	total bilirubin
TC	total cholesterol
TG	triglycerides
TyG	triglyceride-glucose index
UA	uric acid
WBC	white blood cell count

## Appendix A

**Table A1:** Summary of survival events and follow-up in the overall cohort, training set, and validation set.

Dataset	N	Death Events, *n* (%)	Censored, *n* (%)	Median OS, Months	Median Follow-Up, Months	Deaths at 12 m, *n*	Deaths at 24 m, *n*
Overall	134	104 (77.6)	30 (22.4)	15.0	59.7	57	91
Training set	94	76 (80.9)	18 (19.1)	15.1	60.2	38	66
Validation set	40	28 (70.0)	12 (30.0)	13.9	56.9	19	25

**Table A2:** Variance inflation factors (VIFs) of all variables.

Variable	VIF
Sex	1.36
TBIL	1.6
tumor size	1.7
Child–Pugh	1.82
Age	2.01
Number of tumors	2.39
BUN	3.08
HDL-C	3.22
Cirrhosis	3.41
BMI	3.85
Total-protein	3.87
TyG	4.01
WBC	4.15
CREA	4.18
LDH	4.22
NLR	4.36
RBC	4.44
UA	4.52
Platelet	4.6
LDL-C	4.75
ALP	4.93
AFP	4.97
TC	6.49
TG	9.83
PT	16.06
Glucose	23.38
D-dimer	27.67
Hemoglobin	30.72
APTT	34.68
Albumin	48.13
AST	62.62
ALT	68.45
Neutrophil%	73.82
Lymphocyte%	86.63

**Table A3:** Schoenfeld residual test for the proportional hazards assumption in the final combined model.

Variable	Chisq	df	*p*
AFP	2.80	1	0.094
BMI	1.38	1	0.240
HDL-C	1.55	1	0.213
ALP	12.05	1	0.00052
Rad-score	8.67	1	0.00323
GLOBAL	16.15	5	0.00643

**Table A4:** Calibration metrics for the combined model.

Data Set	Time Months	Intercept	Slope
Training set	12	1.072	1.192
Validation set	12	1.222	1.291
Training set	24	1.03	1.044
Validation set	24	1.037	1.262
