# Supplementary material for: AI-Driven Radiomics Assisted Prognostic Modeling for Hepatocellular Carcinoma with Portal Vein Invasion: A Retrospective Study
Source: Biomedicines. 2026 Aug 25;14(9):1894. doi: 10.3390/biomedicines14091894 (PMC13604602; doi:10.3390/biomedicines14091894)
Supplement: Supplementary file 1 [file biomedicines-14-01894-s001.zip › biomedicines-4484370-supplementary.pdf]

All CT scans were performed using 64-detector row scanners of the same model (Brilliance 64, Philips Medical Systems, Eindhoven, Netherlands) following the standard protocol of our institution. Although scans were acquired on multiple machines of this model, the same standardized acquisition and reconstruction parameters were applied consistently throughout the study period (2015–2019). Therefore, no scanner-specific harmonization (e.g., ComBat) was applied.
The contrast agent used was iohexol (300 mg iodine/mL; Bayer Schering Pharma AG, Leverkusen, Germany). The contrast agent was intravenously administered prior to the contrast-enhanced CT scan to a total dose according to the dose-to-weight band (1.5 mL/kg), injected at a constant rate of 2.5–3.0 mL/s using a dual-syringe power injector (Stellant D Dual Syringe, Medrad, Indianola, PA, USA). Scanning delays were 30–35 seconds for the early (arterial) phase and 60–70 seconds for the portal venous phase. Images used for radiomics analysis were acquired from the portal venous phase. The uniform scan parameters were as follows:

**Parameter**	**Value**
Beam pitch	0.891
Tube voltage	120 kVp
Tube current	200 mAs
Detector collimation	0.75 mm
Slice thickness	1.0 mm
Reconstruction increment	5.0 mm
Rotation time	0.42 s
Matrix	512 × 512

Supplementary Table S1. Sensitivity analyses for VIF-removed variables

Variable	HR (95% CI)	P	Training C-index (without / with)	Validation C-index (without / with)
Albumin	1.029 (0.951–1.115)	0.476	0.8306 / 0.8292	0.8077 / 0.8149
AST	1.001 (0.999–1.002)	0.265	0.8306 / 0.8321	0.8077 / 0.8135
ALT	1.001 (0.999–1.003)	0.267	0.8306 / 0.8323	0.8077 / 0.8135
PT	1.027 (0.958–1.102)	0.451	0.8306 / 0.8328	0.8077 / 0.8049
APTT	1.014 (0.989–1.039)	0.264	0.8306 / 0.8328	0.8077 / 0.8049
